# Draft Genome Sequence of *Dactylonectria macrodidyma*, a Plant-Pathogenic Fungus in the *Nectriaceae*

**DOI:** 10.1128/genomeA.00278-15

**Published:** 2015-04-16

**Authors:** Martha Malapi-Wight, Catalina Salgado-Salazar, Jill Demers, Daniel Veltri, Jo Anne Crouch

**Affiliations:** a Systematic Mycology and Microbiology Laboratory, Agricultural Research Service, U.S. Department of Agriculture, Beltsville, Maryland, USA; b Department of Plant Biology and Pathology, Rutgers University, New Brunswick, New Jersey, USA

## Abstract

*Dactylonectria macrodidyma* is part of the *Nectriaceae*, a family containing important plant pathogens. This species possesses the ability to induce disease on grapevine, avocado, and olive. Here, we report the first draft genome of *D. macrodidyma* isolate JAC15-245. The assembled genome was 58 Mbp and contained an estimated 16,454 genes.

## GENOME ANNOUNCEMENT

Genera of fungi with cylindrocarpon-like asexual states, such as *Campylocarpon*, *Dactylonectria*, *Ilyonectria*, and *Neonectria* (*Ascomycota*, *Hypocreales*, *Nectriaceae*), are important pathogens of several herbaceous and woody plants worldwide (1, 2). Species in these genera are unified by phenotypic characters, such as orange to red uniloculate ascomata and cylindrocarpon-like asexual morphs that produce abundant ellipsoidal to ovoid 0−1-septate microconidia, 1–4-septate macroconidia, and intercalary globose chlamydospores (3). Grapevine (*Vitus* spp.) is one of the plants most affected by these fungi, with infection resulting in cankers, root rots, and black foot diseases in nurseries and young plantations. Black foot rot of grapevine is a well-documented disease worldwide, and to date has been associated with fungal species in the four genera previously mentioned (3–5). In recent years, there have been several reports of black foot rot of grapevine caused by *D. macrodidyma* (= *Ilyonectria macrodidyma*) (4, 6). In addition, this pathogen has been reported to induce root rots on avocado (*Persea americana*) (7) and olive (*Olea europaea*) (8) trees. This draft assembly provides the first genomic resource for this destructive plant pathogen and increases the diversity of publically available genome sequences for this important family of fungi.

*Dactylonectria macrodidyma* isolate JAC15-245 genomic DNA was extracted from hyphal tissue using the OmniPrep DNA kit (G-Biosciences, St. Louis, MO, USA) according to the manufacturer’s protocol and subsequently purified using the Zymo Genomic DNA clean and concentrator kit (Zymo Research, Irvine, CA, USA). A dual-indexed library was constructed using the TruSeq DNA PCR-free kit (Illumina, Inc., San Diego, CA, USA), and evaluated for quality and size using the QIAxcel advanced system (Qiagen, Germantown, MD, USA) and the LabChipXT DNA 750 (Caliper Life Sciences, Hopkinton, MA, USA). The library was sequenced on an Illumina MiSeq sequencer (Illumina, Inc.) using a 2 × 300 cycle MiSeq reagent kit version 3 (Illumina, Inc.). Sequence reads were processed and assembled using CLC Genomics Workbench version 7.5.1 (CLC Bio, Boston, MA, USA). After removing adapters and indices, reads were trimmed to remove bases with low Phred quality scores (limit 0.05) and strings of ambiguous bases >2. Reads <15 bp and >600 bp were also discarded. A total of 8,337,788 paired-end reads were assembled using a *k*-mer size of 51 and a bubble size of 50. The genome assembly was 58 Mbp, organized into 850 scaffolds, with an average read depth coverage of 46.66-fold. The assembly was contained in 1,466 contigs (maximum length, 1,533,535 bp; average length, 55,189 bp), with a scaffold *N*_50_ of 336,383 bp and an average G+C content of 49.86%. Gene annotation using the Augustus program (9) with *Fusarium graminearum* gene models predicted a total of 16,454 genes. CEGMA analysis against a conserved set of 248 protein families that occur in eukaryotes (10) determined the assembly of the draft genome to be 96.77% complete.

### Nucleotide sequence accession number.

This whole-genome shotgun project has been deposited in DDBJ/ENA/GenBank under the accession number JYGD00000000. The version described in this paper is the first version.
